# Outlook on Thailand's Genomics and Computational Biology Research and Development

**DOI:** 10.1371/journal.pcbi.1000115

**Published:** 2008-07-25

**Authors:** Wannipha Tongsima, Sissades Tongsima, Prasit Palittapongarnpim

**Affiliations:** 1 National Science and Technology Development Agency (NSTDA), Pathumtani, Thailand; 2 National Center for Genetic Engineering and Biotechnology (BIOTEC), Pathumthani, Thailand; 3 Department of Microbiology, Faculty of Science, Mahidol University, Bangkok, Thailand; University of California San Diego, United States of America

**Box 1. Authors' Biographies**

**Wannipha Tongsima, M.S.**, obtained her master's degree in Industrial Microbiology from Chulalongkorn University, Thailand. She was involved in founding the Bioinformatics research program in BIOTEC. To reinforce the research activity in this area, she also helped organize the first International Conference on Computational Biology (InCoB), held in Bangkok in 2002. Later, she was appointed to manage one of the first BIOTEC ethnic-specific human genetic variation programs, named the Thailand SNP Discovery Project. She works as a Genomic Medicine program coordinator for the Cluster and Program Management Office (CPMO) of the National Science and Technology Development Agency (NSTDA), which is an umbrella organization of four other national research centers in Thailand, including BIOTEC.

**Sissades Tongsima, Ph.D.**, received his doctoral degree in Computer Science and Engineering from the University of Notre Dame, Indiana, United States. He has worked for the National Electronics and Computer Technology Center on High Performance Computing (HPC) and Computational Grid. During 2002–2004, he cochaired the Asia-Pacific Advanced Network (APAN) Grid Working Group. In 2003, he shifted his research direction from HPC architecture to bioinformatics research, when he started working for BIOTEC, and constructed the ThaiSNP database. His main research interest is in developing algorithms and databases for analyzing various research projects on human genetic variation. He currently heads the Genome Institute biostatistics and informatics laboratory at BIOTEC.

**Prasit Palittapongarnpim, M.D.**, earned his medical degree from Mahidol University, Thailand, and his B.S. in Mathematics from Ramkumhang University, Thailand. He is a Fellow of the Royal College of Pediatricians of Thailand and also an Associate Professor in Microbiology at Mahidol University, where he has conducted research focusing on tuberculosis. While holding a Deputy Director position, he initiated the Bioinformatics research program at BIOTEC in 2002 and led the organization of the first InCoB conference in 2002. He is currently a Vice President of NSTDA.

## Introduction

With a wealth of biodiversity, a long tradition of agriculture-based industries, and an established medical and biotechnological research and development community, Thailand has become an attractive location for life sciences investment. The large amount of data generated in many areas of life sciences requires visualization, management, and analysis, principally through bioinformatics. To become successful, Thailand's research community should emphasize establishing core technologies, such as genomics and bioinformatics, to boost development of agriculture, food processing, and biomedical research. The Thai government realized the importance of this field and created a national policy to greatly increase Thailand's participation in bioinformatics and genomics, budgeting for specific development goals in research infrastructure, education, and sustainable human resources.

Thailand has not lagged behind in bioinformatics research activity and recognizes the importance of bioinformatics through increased policy awareness, human resources development, and increased research activity involving genomic-scale data generation and computational analyses. Many applications of genomics and bioinformatics to biomedical research and development in Thailand have progressed substantially during the past few years, leading to successful applications in some specific local areas. However, the applications to other important areas, such as agriculture, are hampered by the limited availability of genomic sequence data and the lack of necessary biochemical/physiological information. With the advent of more and more genomic information in public databases, Thailand's research community is striving to adopt comparative genomics to obtain information of direct relevance to the country's health and industrial needs. This article highlights Thailand's contribution to genomics and bioinformatics in the following areas: (1) policy support from the Thai government, (2) capacity building through infrastructure/education/human resources, and (3) research and development in genomics and computational biology. (See Box 1 for Authors' Biographies).

## Support through National Policies

Thailand's unique ecosystems are located in several climatic zones: from the temperate north, the rich central plain, the hot and arid northeast plain, to the rainforests in the south with rich mangrove forests along the coastal areas. The Thai government realized the advantages of such biodiversity and founded the National Center for Genetic Engineering and Biotechnology (BIOTEC) in 1983 to foster biotechnology industries. In 2002, BIOTEC moved to the new Thailand Science Park situated in Pathumthani (northern vicinity of Bangkok). To support research and development needs for emerging biotechnology businesses and to become a regional training hub for biotechnology and life sciences development, the Thai government allocated approximately US$16.5 million to set up Thailand's first BioPark within the Thailand Science Park. The National Biotechnology Policy Framework (2004–2009) was established to create opportunities to invest and conduct world-class business and research in Thailand.

Bioinformatics and genomics have been recognized by the country's leaders as key priority technological disciplines. Therefore, the applications of both disciplines in biomedical and agricultural research have been enthusiastically endorsed and financially supported. The Thailand Board of Investment (BOI) promotes foreign investment in bioinformatics-related business located within the BioPark through corporate tax exemption for up to eight years. Current businesses with foreign investment include bioinformatics solution service providers, sequencing services, and genotype testing services. In 2004, the Thai government, with royal decree, established the Thailand Center of Excellence for Life Sciences (TCELS) with the aim of supporting investment and development in life sciences business by creating partnerships with foreign investors. To promote life sciences business, TCELS receives research funding from the Thai government as well as from other business-related sources. To protect the investment of local and foreign researchers, TCELS also promotes legal protection of science discoveries and innovations.

## Promoting Bioinformatics

Efforts have been made to promote awareness of bioinformatics in Thailand, such as organization of the first International Conference on Bioinformatics (InCoB2002): North South Networking, by BIOTEC in collaboration with the Asia-Pacific Bioinformatics Network (AP-BioNet). This conference invited a number of distinguished speakers, including Dr. Carlos Morel, the Director of the special program for Research and Training in Tropical Diseases (TDR) of the World Health Organization (WHO) at the time. His influential role succeeded in persuading many senior executives in the Thai scientific community to realize the importance of genomics and bioinformatics. Dr. Michael Waterman brought to the attention of local computer scientists the need to embrace computational biology challenges. Other invited scientists demonstrated how useful bioinformatics is, especially in the postgenomic era. Following the success of this meeting, and with AP-BIONET coordination, InCoB has now become an annual event organized mostly by developing countries. The 2007 meeting was held in Hong Kong, China. With support from the aforementioned national policies, the Thai government invests approximately US$5 million per year to promote activities in bioinformatics and computational biology through (1) research and development, (2) improving genomics and bioinformatics infrastructure, (3) supporting bioinformatic education, and (4) developing a sustainable human resource program. The following subsections discuss the last three supports in more detail.

### 

#### Infrastructure

In the early days of the Internet, Thailand had a poor connection. To alleviate the network bottleneck, Thailand became part of the Bio-Mirror network [1], which is a collaboration between AP-BIONET and IUBio-Archive (a portal of biology data and software founded in 1989 by Indiana University's Genome Informatics Lab). The Bio-Mirror in Thailand (http://bio-mirror.ku.ac.th) aims to provide local access to various public databases, e.g., GenBank. Currently, the networking infrastructure has been dramatically improved with two major governmental Research and Educational Networks (RENs), namely UniNET for local universities and ThaiSARN 3 for national research institutes. In 2006, the Software Industry Promotion Agency (SIPA), under the Ministry of Information and Communication Technology, funded US$1.5 million for the installation of the largest computational grid infrastructure in Thailand to support all kinds of research in computational sciences. BIOTEC has also supported computational life sciences research by purchasing a series of high performance computers (HPC) since 2002. In late 2008, BIOTEC plans to purchase a cluster computer system with the performance of seven terra floating point operation per second (TFLOPs). To date, BIOTEC has allocated more than US$1.7 million to improving bioinformatic and genomic computing infrastructures. To strengthen its research capabilities, BIOTEC founded the BIOTEC Genome Institute, investing US$2.5 million for a state-of-the-art pyrosequencer, called 454 GS-FLX, and a matrix-assisted laser desorption/ionization time-of-flight mass spectrometer (MALDI-TOF) system.

#### Education

Realizing that the use of genomics and bioinformatics will facilitate the cash-starved research on tropical and neglected diseases, TDR–WHO initiated a program in 2003 to further bioinformatics expertise in developing countries. Four training centers were founded around the world to offer bioinformatics-related courses to local trainers in developing countries. The Center for Bioinformatics and Applied Genomics (CBAG), Mahidol University, (http://www.ssi-tdr.net/cbag/), is one of the centers that provides regular training courses by instructors from many renowned institutes around the world. Upon the completion of the training, the local trainees are expected to use the knowledge and skills in their research.

The National Science and Technology Development Agency (NSTDA) initiated an introductory online course on bioinformatics, distributed through an e-learning program (http://biohpc.learn.in.th/) in 2003 for development of bioinformatics personnel. This course was designed by researchers from various institutions for graduate and postgraduate students throughout the country. Thai universities can incorporate this e-learning program into various graduate-level curricula in life sciences. Hence, this activity could jump-start bioinformatic education in Thailand, where local bioinformatics experts are still very few in number. Subsequently, when more experienced bioinformaticians become available, more universities will start offering their own courses.

In 2004, the King Mongkut University of Technology Thonburi (KMUTT) started the first Master's program in Thailand on bioinformatics with student scholarships supported by BIOTEC (http://www.bioinformatics.kmutt.ac.th/course.php). This Master's program accepts 10–15 students per year from a wide range of scientific backgrounds, who are required to complete two internships in either national or international research institutes. By the end of 2007, this program had produced 34 bioinformaticians. The majority are now employed in local research institutes, while some have pursued doctoral degrees abroad in bioinformatics and in computational biology. In 2008, KMUTT along with two other leading Thai universities, Kasetsart and Mahidol, will be offering doctoral programs on bioinformatics. These programs are expected to produce five or more bioinformatics Ph.D.s combined each year.

#### Human resources

Despite the growth in bioinformatics education as described above, there are still few Thai researchers in this field. Currently, 40 or fewer Thai researchers with Ph.D.s from various fields, e.g., mathematics, chemistry, computer sciences, and biology, work in the area of bioinformatics and computational biology. The majority of these researchers received their doctoral degrees abroad, with scholarship support from the Thai government. The scholarships are awarded on the condition that the graduates return to work in Thailand, and it is expected that 20 or more Thais currently abroad will graduate in bioinformatics/computational biology in the next five years. In the future, it is anticipated that doctoral level programs in bioinformatics will be offered in Thailand to promote sustainable development and management of bioinformatic human resources.

## Genomic Data Generation

During the past ten years, Thai researchers together with collaborators in other countries have strived to master various genomic technologies, including DNA sequencing, expression profiling, and systems biology. This section discusses Thailand and its contributions to the generation and utilization of genomic data ranging from small organisms, such as viruses and bacteria, to complex organisms, such as rice and cassava.

### 

#### Whole genome sequencing projects

*Burkholderia pseudomallei*, the causative bacterium of melioidosis, is the first organism to be whole-genome sequenced by Thai scientists. This gram-negative bacterium is a soil saprophyte in melioidosis endemic areas, particularly Southeast Asia. It is responsible for 20% of acquired septicemia cases in northeastern Thailand, with an approximately 50% fatality rate. In 1998, the 7.25 Mb genome of *B. pseudomallei* K96243 was sequenced by a research team at the Wellcome Trust Sanger Institute, with significant contribution from Dr. Sirirurg Songsivilai, Mahidol University [2]. The relatively large genome contains 16 genomic islands that together make up 6.1% of the entire genome. The genes in the genomic islands are absent from related organisms such as *B. mallei* and may account for the clinical features of melioidosis caused by the organism. More information and the recent progress of the research on the bacterium were recently reported [3].

With rice as a staple food plant, a Thai research team led by Dr. Apichart Vanavichit, the director of BIOTEC and Kasetsart University's joint Rice Gene Discovery Unit, joined the International Rice Genomic Sequencing Project (IRGSP) to sequence its genome. From this news event, the Thai press stimulated public interest in genomics and bioinformatics, leading to greater public awareness of the feasibility and potential of these two disciplines. Subsequently, the Thai researchers sequenced two million base pairs from rice (*Oryza sativa* spp. japonica) Chromosome 9 [4],[5], an activity that has fostered the ability of Thai researchers to obtain and utilize large amounts of genomic information.

The dramatic decrease of the DNA sequencing cost over the past few years has allowed Thai researchers to employ the technology to sequence small genomes of organisms important for local research questions. Avian influenza was inevitably chosen due to its impact on Thailand and the rest of the world, and to help solve the recent dispute regarding the sharing of the viral samples between the affected developing and developed countries [6]. The sequence information is important for evaluating control measures, such as vaccines or drugs, and for monitoring the genetic changes of the circulating avian influenza virus. The sequence information could also answer basic and epidemiological questions and trace spreading pathways of the virus [7]–[9].

At the same time, other viruses, such as dengue viruses [10],[11] and viruses of agricultural importance [12], have also been sequenced. Such information provides insight into the evolution of these viruses and pathophysiological understanding of infectious diseases. For example, the genomic sequences of dengue virus type I collected over a 30-y period revealed the associations between genetic diversity and increase in the serotype prevalence, and decline in serotype prevalence with clade replacement [13]. The expertise gained from working with these viruses allows Thailand to effectively utilize genomic sequencing to cope with future emerging viruses.

Recently, BIOTEC invested in a 454 GS-FLX pyrosequencer and used it together with the conventional Sanger method to de novo sequence the *Spirulina platensis* cyanobacterial genome. Led by Drs. Supapon Cheevadhanarak and Somvong Tragoonrung, this sequencing project aims to increase understanding of this organism's metabolic and regulatory pathways, accelerating research and development of *Spirulina* for commercial purposes. The project is in the finishing steps, and the results should soon be available to the scientific community.

#### Thailand SNP discovery

Following the release of the International HapMap data, efforts were made by Thai researchers to apply the information to improve medical practices and health. The first research question was whether known SNPs were an adequate representation of the Thai population or not. A collaborative project between BIOTEC, Mahidol University, Institut Pasteur, and the Centre National de Génotypage (CNG) [14] in Evry, France, was initiated to collect intragenic SNP markers from 194 candidate genes of 32 healthy Thai volunteers' DNA samples (randomly chosen from 64 volunteers whose profiles fit the selection criteria). As of January 2008, 25% (876 SNPs) of the 3,523 discovered SNPs were novel when compared with SNPs reported in the dbSNP database. The novel SNPs, however, tend to have low frequency (70% of novel SNPs have allele frequencies less than 5%) and may not be very useful for a large-scale disease-association study in Thailand. However, the results may help to locate disease-predisposing genes and prompt an evaluation of the need to resequence the genes in the Thai population. A whole genome resequencing project at this point would still be exorbitant. In the near future, the price of genome sequencing may drop low enough for a whole genome sequencing of Thais to be feasible.

The Asian populations included in the International HapMap project were Chinese and Japanese. The Thai population is likely to be more diverse in origin and has a significant additional genetic relationship with the Indian population, among others. Dr. Surakameth Mahasirimongkol, (Department of Medical Sciences, Ministry of Public Health) and Dr. Yusuke Nakamura, (SNP Research Center at RIKEN and the Human Genome Center at the University of Tokyo) were supported by TCELS to study the transferability (from the Japanese population to the Thai population) of 861 haplotype-tagging SNPs (htSNPs) in 166 drug-related genes by genotyping 280 individuals from four regions in Thailand (north, central, northeast, and south). It was concluded that amongst these genes, the allele frequencies of all four Thai regional populations are generally similar to each other and to the Japanese htSNPs. The study demonstrates that the htSNPs from the Japanese population in the HapMap database are very useful in selecting SNPs to be genotyped in case/control association studies in Thailand [15]. The transferability could probably be extended to other genes as well.

In order to facilitate medical genetic scientists, the BIOTEC ThaiSNP database has collected SNPs from the aforementioned SNP studies as well as from large-scale SNP genotyping projects (see http://thaisnp.biotec.or.th/). The database allows search for Thai-specific SNPs as well as other ethnic SNPs reported in the public domain dbSNP from the National Center for Biotechnology Information (NCBI). Moreover, SNP locations from different populations can be displayed in a comparative view illustrating the underlying differences. Researchers can select SNPs that are only polymorphic in the Thai population and design specific primers to genotype such SNPs. To assist this process, ThaiSNP also provides a customized Primer3 program to design allele-specific primers [16] as well as resequencing primers. All the aforementioned activities have been fostered by a local human genetic consortium supported by BIOTEC.

#### High-throughput genotyping

Further studies have been conducted to assess genome-wide SNP allele frequencies of the Thai population from various disease-association studies. Most of them utilized parallel genotyping techniques such as MALDI-TOF Mass Array and Affymetrix array. The first of such studies, funded by the United States National Institutes of Health (US NIH), is an identification of the genetic determinants that would affect the severity of β-thalassemia/HbE diseases, conducted by Dr. Suthat Foocharoen, Mahidol University, in collaboration with SEQUENOM and Boston University. β-thalassemia/HbE is a common blood disorder in Southeast Asia, manifested as reduced normal and abnormal β-globin, caused by a combination of genetic variants. The allele frequencies of approximately 100,000 SNPs amongst 400 β-thalassemic patients with either mild or severe symptoms were determined by a Mass ARRAY System. The SNPs associated with disease severity have been identified and are being verified. Allele frequencies across a large number of known SNPs were also obtained, which may be useful for other research studies.

The study led by Dr. Boonsong Ongphiphadhanakul, Mahidol University, sought to identify the genetic factors affecting the severity of osteoporosis. This study utilized Affymetrix SNP array for genotyping pooled DNA, and therefore provided allele frequency of a different set of known SNPs. Another study aimed to identify genes associated with adverse drug reactions to nevirapine, which is one of the first-line drugs against HIV. GPO-VIR is a local anti-retrovirus drug produced by the Thai Government Pharmaceutical Organization (GPO) as a combination of nevirapine, zidovudine, and lamivudine. This drug has been produced since December 2001 and is listed in the National List of Essential Medicines. However, adverse drug reactions, particularly in the form of drug rash, occur very commonly (32%–48%) in nevirapine-prescribed individuals. The potentially lethal form, Stevens-Johnson syndrome, occurs in 0.5%–1% of people. The adverse drug reactions would inevitably force the people to use much more expensive drugs [17]. In collaboration with Dr. Nakamura, the genome-wide SNPs of 80 individuals with drug rash caused by nevirapine and the control of 80 individuals without drug rash are being compared, with the expectation that some SNP biomarkers can be identified as clinically useful predictors of such adverse drug reactions.

Dr. Nakamura also collaborates with TCELS to discover the genes associated with post-traumatic stress disorder found in Thai individuals who experienced the great Asian tsunami in 2004 [18]. It should be noted that this was one of those rare occasions in which a large number of people were simultaneously exposed to the same traumatic experience. Moreover, similar collaborations are in place with the Department of Medical Sciences, targeting other medical-related projects, including leprosy, leukemia, hepatocellular carcinoma, and Leber hereditary optic neuropathy (LHON).

#### EST sequencing

Expressed sequence tags (ESTs) are studied to identify genes from agriculturally important organisms, many genomes of which are still uncharacterized. The black tiger shrimp (*Penaeus monodon*) and cassava (*Manihot esculent Crantz*) are two agriculturally important species that Thailand has put efforts toward to obtain comprehensive EST libraries.

Currently, more than 40,000 ESTs of the black tiger shrimp have been sequenced [19], and more than 10,000 unique gene fragments have been identified. The EST libraries were constructed from RNA extracted from various tissues such as eye, leukocyte, testis, and ovary. From these data, DNA markers [20] such as microsatellites [21] and SNPs have been discovered. Some important genes, such as antimicrobial peptides [22], host–defense related genes [23], fortilin [24],[25], and sex-related genes [26], were reported. The EST sequence information is incorporated into the Black Tiger Shrimp EST Database (http://pmonodon.biotec.or.th/database.jsp), which may also assist future commercial domestication of various aquatic invertebrates, including other species of shrimps, lobsters, and crabs.

Cassava is a staple food plant in many countries in Africa as well as being of utmost importance for industry in Asia, where it is a major source of animal feed as well as a potential biomass for cost effectively generating ethanol [27]. To understand this important organism, BIOTEC and the Nara Institute of Science and Technology (Japan) have collaborated in sequencing approximately 100,000 ESTs from 12 leaf and root libraries. The EST sequences will be useful for comparison with *Arabidopsis*, a dicotyledonous species related to cassava.

#### Proteomics

2-D gel protein electrophoresis is an established experimental tool in several Thai laboratories and has been used to identify plant and animal proteins expressed in various conditions, including cassava, peanut, shrimp [28], and microbes, such as *B. pseudomallei* [29], *Bacillus stearothermophilus* [30],[31], *Spirulina*, and malarial parasites. Proteomic analysis has also been applied to biomedical research on various kinds of samples, including a cholangiocarcinoma cell line [32],[33], but in the main on urinary samples. It is hoped that proteomic profiling of urine will lead to better understanding of renal physiology, several disease mechanisms, and identification of novel biomarkers and therapeutic targets [34]. It has been proven particularly useful in identifying protein changes following chronic potassium depletion, a condition that leads to skeletal, muscular, and kidney damage and is relatively common among Thais [35],[36]. Other proteomic studies by Thai investigators include renal damage in diabetes mellitus [37] and children with Hodgkin's lymphoma and IgA nephropathy [38].

#### Microarray and systems biology

A widely used method for studying gene expression is by measuring mRNA abundance by micro- or macroarray hybridization. This method has been used by Thai researchers to study drug mechanism in tuberculosis [39], pathogenesis of dengue infections, nasopharyngeal carcinoma [40], and cholangiocarcinoma [41],[42]. Methods for analysis of these data have also been developed [43].

Different levels of information, such as genomics and microarray gene expressions, have constantly been generated by research institutes around the world. Systems biology is a field which studies metabolic and regulatory network profiles by utilizing various in silico tools to reconstruct a biological system from miscellaneous genomics data, such as sequences, RNA and protein expressions, and metabolite concentrations. The areas of Thai topical interest include starch biosynthesis pertaining to cassava, the lipid synthetic pathway of *Spirulina* and yeasts, as well as the core metabolic pathways of malaria and tuberculosis [44].

## Bioinformatics Development and Data Utilization

As the cost of genomic research decreases and more whole genome-scale research projects are completed, many researchers in Thailand have adopted various computational biological methods to analyze the large amounts of genomic data to generate biological hypotheses, which can be subsequently validated by “wet” laboratory experiments.

### 

#### Identification and application of DNA repeats

Genomes contain microsatellites, or numerous short segments of tandemly repeating sequences two to five nucleotides long. In humans, disease manifestations can be associated with microsatellite polymorphisms. For example, a tandem repeat in the nitric oxide synthase promoter was found to be associated with severe malaria in Thailand [45]. Microsatellites are also exploited to identify genetic relationships for forensic applications.

In plants and animals, a microsatellite that is linked to a gene locus of interest, usually called a simple sequence repeat (SSR) marker, is used to assist selective breeding programs. Marker-assisted selection obviates the need for expensive and laborious phenotypic testing. It also allows selection at an early stage of growth before the phenotype of interest is observable.

When a complete genome of an organism is available, various bioinformatic tools can be used to rapidly identify putative SSR loci. Even when a genome is not yet completely sequenced, as in the case of most organisms of agricultural importance, the candidate SSR markers can still be discovered from EST sequences, which may contain other forms of meaningful repeats. Thai scientists have identified candidate SSRs from cassava, sugarcane (*Saccharum L.*), peanut (*Arachis hypogaea* L.), oil palm (*Elaeis guineensis*, Jacq.), soybean (*Glycine max* Merr.) [46], and rubber tree (*Hevea brasiliensis* Muell. Arg.) [47]. It is anticipated that these and many more SSR markers will be useful for selective breeding programs.

Prokaryote genomes also contain tandem repeats, albeit much less frequently and usually with longer unit length than eukaryotes. The repeats may be polymorphic and can be used for evaluating genetic relationships between strains of microbes. Early Thai research efforts led to the discovery of variable number tandem repeats (VNTR) in the genome of *Mycobacterium tuberculosis* [48], which were later shown to be useful markers for epidemiological studies [49]. Tandem repeats have also been found in many bacterial species, including *Escherichia coli*, *Salmonella*, *Shigella*, *Vibrio cholerae*, *Leptospira*, and non-tuberculous mycobacteria. The usefulness of such repeats for epidemiological studies is being evaluated [50].

#### Threshing the rice genome

With the completion of the rice genome sequence, a Thai Rice Genomics database, dubbed “RiceGeneThresher” (http://rice.kps.ku.ac.th/cgi-bin/GeneThresher), was created. It contains DNA sequences, gene information, and QTL mapping information from a variety of rice species. Postgenomic research has begun to identify genes associated with agronomic traits such as cooking quality [51], submergence tolerance [52]–[54], drought tolerance, brown planthopper (*Nilaparvata lugens* Stal) resistance [55], leaf and neck blast resistance [56], and other traits [57]. The most notable discovery was of the aroma mechanism of Thai jasmine rice [58], which is locally prized as a national asset. Genetic markers associated with aroma and submergence tolerance have been filed for international patents, and can be utilized for rice breeding programs. Furthermore, knowledge of the genetic mechanisms governing these properties is being exploited to conduct similar research in other cereals.

#### Utilization of SNP data in biomedical research

Thailand is among the few countries in the world to provide universal health coverage for all citizens. Diagnoses that can predict occurrence or progression of diseases are needed to minimize the cost of health care. Advances in genomics have led to the discovery of various biomarkers, although the clinical usefulness of most of them is yet to be confirmed. Hepatitis B infection is widespread in Thailand, and, as such, chronic hepatitis followed by hepatocellular carcinoma (HCC), a liver cancer, is common. Genomic research in this area uncovered SNPs in the interleukin-18 (IL-18) gene and its promoter, which were associated with chronic hepatitis and development of HCC among chronic hepatitis B patients, respectively [59],[60]. Moreover, it was demonstrated that HCC prognosis could be assessed by serum IL-18 level [61] and the methylation status of LINE-1 repetitive sequences in genomic DNA derived from sera [62].

The ability to predict the prognosis of cancer patients is of particular interest since it may guide how aggressive the treatment should be, while minimizing the side effects. Similar studies have, therefore, been done with other cancers. Markers for long-term survival have been identified for cholangiocarcinoma, another common liver cancer in Thailand [63]. For oral cancer, a mutation associated with recurrence has been identified [64].

#### Drug target discovery

The levels of antibiotic resistance of some medically important microbes have reached an alarming level, exemplified by the emergence of extremely drug-resistant tuberculosis as well as drug-resistant malaria. Most antibiotics bind specifically to target proteins and disrupt their functions, leading to bacterial cell death or growth arrest. Current antibiotic targets include only a few dozen proteins in contrast to the hundreds of possible targets.

Dr. Yongyuth Yuthavong and his team have focused on identifying antimalarial targets [65] and developing test methods based on folate metabolism [66],[67]. This pathway provides two targets for current antimalarials: dihydrofolate reductase (DHFR) and dihydropteroate synthase (DHPS). Other enzymes of interest as drug targets in the pathway include thymidylate synthase, an enzyme naturally fused with DHFR in the malarial parasites, serine hydroxymethyltransferase, methylene tetrahydrofolate dehydrogenase, and methionine synthase.

Other work focuses on comparative genomics of bacteria. For example, based on the concept of a minimal gene set needed for bacteria in order to perform the core processes of life [68], a number of genes likely to be essential for *M. tuberculosis* have been identified. Among them, 47 genes have no human orthologs, making them theoretically safe as drug targets. Antisense mutagenesis has demonstrated the essentiality of the gene coding for fructose-1, 6-bisphosphate aldolase. Compounds known to inhibit the enzymes in *E. coli*, as well as their derivatives, were tested against *M. tuberculosis*. One compound, 5-chloro-8-hydroxyquinoline, was found to be active against laboratory and clinical strains of the bacterium [69]. Ongoing work includes many other target proteins that are amenable to formulation of target-based screening.

#### Drug candidate discovery

Once the drug targets are known, the next logical step is to identify drug candidates. Conventionally, this is done by screening thousands of compounds against target proteins or target organisms. The hit rate is usually low. Therefore, a large library of compounds is usually needed. The screening can also be done in silico. A combined docking and neural network approach was developed by a Thai team to screen anti-HIV-1 inhibitors for two targets, HIV-1 reverse transcriptase and HIV-1 protease [70],[71]. A similar approach has been applied to identify possible herbal compounds that can dock to the avian influenza neuraminidase target. A number of these compounds with possible inhibitory activity against the protein were identified. The predictions are being validated in Thai laboratories. In addition, structurally based drug designs, particularly against the target DHFR protein of the malarial parasite *Plasmodium*, are particularly fruitful, as previously reviewed [72]. The success of this research is exemplified by the recent issue of a US patent on pyrimidine derivatives that inhibit this enzyme [73]. A similar strategy has also been applied to other microbes, namely *M. tuberculosis* and dengue virus.

## Future Prospects

With an increasing number of well-trained scientists in the field of genomics and bioinformatics, Thailand should be able to keep up with the advances in life sciences research areas. Expansion of research and development utilizing bioinformatics is needed to solve local agricultural and biomedical problems. With the endorsement of the Thai government and funding system, computing facilities and other genomic platforms should be able to meet the demands of the Thai research community. These establishments recognize the utmost importance of collaboration between Thai molecular and computational biologists so that they can share problems and expertise to gain new biological insights. The application to agricultural biotechnology is particularly challenging. With the lack of genome sequence data and basic biological information on agriculturally important organisms, progress in this field could be impeded. The main challenge of comparative genomics in Thailand and other developing countries is, therefore, to make inferences about organisms of local interest from well-studied organisms.
